# All-inside technique in ACL reconstruction: mid-term clinical outcomes and comparison with AM technique (Hamstrings and BpTB grafts)

**DOI:** 10.1007/s00590-020-02798-w

**Published:** 2020-09-16

**Authors:** Andrea Pautasso, Marcello Capella, Luca Barberis, Luca Drocco, Riccardo Giai Via, Alessandro Bistolfi, Alessandro Massè, Luigi Sabatini

**Affiliations:** 1 University of Turin, Via Gianfranco Zuretti 29, 10126 Turin, Italy; 2 Orthopaedic and Traumatology Department, Orthopaedic and Trauma Center, Città della Salute e della Scienza, University of Turin, Via Gianfranco Zuretti 29, 10126 Turin, Italy

**Keywords:** ACL reconstruction, Hamstrings, Bone-patellar tendon-bone, All-inside technique, Antero-medial technique

## Abstract

**Purpose:**

The aim of this study was to define the subjective and objective clinical results of all-inside surgical technique at a medium-term follow-up and to compare these results with those obtained from antero-medial (AM) ACL reconstruction technique using hamstrings (HS) or bone-patellar tendon-bone (BpTB) grafts to detect eventual superiority of one technique to another.

**Methods:**

A retrospective analysis of routinely collected data was conducted. Inclusion criteria were ACL reconstruction through all-inside technique or AM technique with HS or BpTB performed between January 2015 and May 2018; age between 15 and 30 year old; minimum 24 months’ available follow-up. Exclusion criteria were contralateral ACL reconstruction; need for any other associated procedures during surgery. Clinical outcomes were assessed with KOOS, Lysholm, Tegner scores and KT-1000 device.

**Results:**

According to the selection criteria, 157 patients were enrolled and divided subsequently into 3 groups: all-inside (51 patients), AM-HS (53 patients) and AM-BpTB (53 patients). A significant postoperative improvement of each score in all groups was detected. The mean KT-1000 was 3.1 ± 1.0 mm in all-inside group, while 3.3 ± 1.4 mm and 2.5 ± 0.4 mm in AM-HS and AM-BpTB groups, respectively. Comparing the results obtained, no statistically significant difference was found between the three techniques (*p* = 0.27). Statistically significant differences were highlighted in surgical duration: all-inside method was the longest (117′), followed by AM-BpTB surgery (101′) and AM-HS technique (87′).

**Conclusions:**

The all-inside technique showed good postoperative results at medium-term follow-up. It could be a valuable solution for ACL reconstruction, especially in young patients due to its less invasiveness, despite surgical skills and time needed.

**Levels of evidence:**

Level IV.

## Introduction

Anterior cruciate ligament (ACL) surgical reconstruction dates back to the late nineteenth century when Dr. Robson performed the first successful ACL repair on a 41-year’s old coal miner [1, 2]. Over the years, the techniques were changed and improved, becoming less invasive. The greatest innovation occurred in the eighties with the introduction of arthroscopy [3]. From that moment, the technique was further modified and optimized in order to overcome the open procedure making the reconstruction less invasive, lowering complications, intra-operative bleeding and postoperative pain to provide quick recovery [3]. Following and implementing these principles, the all-inside reconstruction technique was conceived [4, 5].

The all-inside technique for ACL reconstruction [5], compared to the traditional Antero-Medial (AM) or Transtibial methods, features substantial improvements including two closed-socket tunnels, double (femoral and tibial) suspensory fixation and smaller skin incisions [6]. That allows the graft insertion to be performed through an arthroscopic portal, minimizing postoperative bleeding, soft tissue damage and especially bone loss (reduction from 54 to 64%) and postoperative pain [7–9].

Because of this, all-inside technique, side by side with all-epiphyseal and hybrid techniques may be a valuable option for younger patients with open growth plate in order to preserve and guarantee a physiological skeletal growth [10].

Moreover, according to recent studies [11–13] the all-inside and the AM techniques ensure more anatomical femoral tunnel placement if compared to the transtibial technique, which is mandatory to obtain good clinical outcomes and lower the graft failure risk [14]. Looking further, the AM technique, despite its flexibility due to the independent femoral and tibial tunnels placement, could be technically demanding and often leads to a very short femoral tunnel with potentially unstable button fixation, whereas the all-inside technique enables a more precise and anatomical-oriented femoral tunnel drilling, straight to the ACL footprint as recommended in the literature [11, 13, 15, 16].

All-inside surgical technique, because of the sockets obtained by retrograde drilling, requires shorter prepared graft (5–7 cm against 11–13 cm of the standard methods [7, 17, 18]) so allows to harvest only the semitendinosus tendon, saving the gracilis muscle. The classic bone-patellar tendon-bone (BpTB) autograft can also be used with this surgical technique, flipping the bone plug at one side as described [19].

In this context, the aim of this study was to define the subjective and objective clinical results of all-inside surgical technique at a medium-term follow-up and to compare these results with those obtained from antero-medial (AM) ACL reconstruction technique using hamstrings (HS) or bone-patellar tendon-bone (BpTB) grafts to detect eventual superiority of one technique to another.

## Material and methods

A retrospective monocentric (CTO Hospital—Città della Salute e della Scienza, Turin (Italy)) analysis of patients aged between 15 and 30 years old with diagnosis of ACL injury who underwent arthroscopic reconstruction from January 2015 to May 2018 at was conducted.

The inclusion criteria were: ACL injury, age range at surgery of 15–30 years old; The exclusion criteria were: multiligamentous knee instability; associated meniscal injuries or cartilaginous lesions (Outerbridge < 2); homologous or synthetic tendons grafts choice; previous controlateral ACL reconstruction; ACL reconstruction failed surgical revision; concomitant antero-lateral ligament reconstruction or lateral tenodesis (e.g., Arnold-Coker) for residual rotatory knee instability (e.g., pivot-shift grade 2 or more, antero-lateral ligament disruption, Segond fracture, etc.); postoperative follow-up less than 24 months.

ACL reconstructions were performed using three different techniques: all-inside surgical technique using a double button suspension system (GraftLink® All-Inside ACL Reconstruction with ACL TightRope® RT and TightRope® ABS—Arthrex©, Naples, FL, U.S.) for a four strands Semitendinosus autograft; AM technique using hamstrings (HS) and GraftMax™ Button ALB (ConMed Corporation, Largo, FL, U.S.) on the femoral side and Genesys™ Matryx® Interference Screw (ConMed Corporation, Largo, FL, U.S.) on the tibial side; AM technique using bone-patellar tendon-bone (BpTB), with GraftMax™ Button BTB (ConMed Corporation, Largo, FL, U.S.) on the femoral side and Genesys™ Matryx® Interference Screw (ConMed Corporation, Largo, FL, U.S.) on the tibial side. All surgical ACL reconstruction was performed by two senior surgeons [LS, LD].

At our institution, the surgical technique was routinely chosen based on age (20 years old or lower) for all-inside technique, while for patients older than 20 years, HS or BpTB harvesting was selected based on a multifactorial decision-making including sports and working activity, characteristics of predicted grafts such as HS diameter and BpTB length and preference of the patient.

We collected the following data: age at surgery, sex, pre-injury sport and the type of trauma that led to ACL disruption. Furthermore, knee function during daily and sports activities, perceived quality of life and return to sport activity were evaluated through specific questionnaires (Knee Injury and Osteoarthritis Outcome Score [KOOS], Lysholm Knee Questionnaire and Tegner activity scale) submitted to the 3 groups before surgery and at the follow-up. Moreover, postoperative physical examination at follow-up was performed by the same operator [AP] including knee laxity evaluation with KT-1000® instrument (MEDmetric, San Diego, CA, U.S.). For each knee, three different measurements were performed; measurements were also taken in the contralateral knee to better identify abnormalities or differences with the native knee. The measurements were taken by exerting a postero-anterior force of 89 N, thus recording the value of the emission of the second sound.

All-inside ACL reconstruction was performed as described by Cerulli et al. [7]. The surgical procedure could be divided in two key part: the semitendinosus harvest and the arthroscopic reconstruction time. We harvested the semitendinosus graft through a mini-open approach with a tendon stripper. The required graft length is 6/6.5 cm; in all the 51 patients, the harvested tendon’s length was longer than 25 cm, so it was possible to fourfold it. During the arthroscopic reconstruction, a 90° femoral aimer (Femoral ACL Marking Hook for RetroConstruction Drill Guide®—Arthrex©, Naples, FL, U.S.) and a 50° tibial aimer (Tibial ACL Marking Hook for RetroConstruction Drill Guide®—Arthrex©, Naples, FL, U.S.) were pointed to the anatomical ACL footprints. The retrograde sockets using FlipCutter® III Drill (Arthrex©, Naples, FL, U.S.) measured about 2 cm. At the end of the procedure, the surgical times were recorded, and the same was done during ACL reconstruction with AM techniques.

Postoperative protocol for ACL reconstruction was the same for every group, consistent with the main guidelines in the literature [20–22].

### Statistical methodology

Data collected were analyzed using IBM SPSS® (Data Analysis and Statistical Software): a Kolmogorov–Smirnov test of normality was used to study the values distribution in all data series. Except for the recorded surgical times, all series of values had a non-normal distribution; therefore, Wilcoxon Signed-Rank test was applied to compare preoperative and follow-up values of each score. Moreover, Kruskal–Wallis test for independent measures was used to compare the different results between the surgical techniques, while one-way ANOVA test for independent measures (and post hoc correction tests like Tukey’s HSD, Scheffé, Bonferroni and Holm) was used to compare the different surgical times. The significance was set at *p* < 0.05.

## Results

According to the inclusion and exclusion criteria, 157 patients were enrolled: 51 patients operated with all-inside method; 53 patients treated with the AM method using hamstrings autograft; 53 patients operated with the AM technique using bone-patellar tendon-bone autograft. The gender distribution was 114 males and 43 females. Mean age at the surgery was 23.4 ± 5.2 (16 – 30) years old in the examined population, while it was 18.1 ± 1.1 years in the all-inside group, 23.0 ± 2.3 years in the AM-HS group and 25.3 ± 3.5 years in AM-BpTB group. The means of follow-up ranged from 36.5 ± 8.4 months in all-inside group to 41.2 ± 14.2 months of AM-BpTB group (all the sample’s features are reported in Table 1).

**Table 1:** Table 1 Description of the sample (AM: antero-medial)

	All-inside	AM with hamstrings	AM with bone-patellar tendon-bone
Number of patients	51	53	53
Gender			
M	33	38	43
F	18	15	10
Mean age at surgery time (years old)	18.1 ± 1.1 (16–20)	23.0 ± 2.3 (21–30)	25.3 ± 3.5 (21–30)
Mean surgical time (min)	117 ± 23 (70–180)	101 ± 21 (50–155)	87 ± 22 (55–140)
Mean age at follow-up (years old)	21.5 ± 1.6 (18–24)	26.6 ± 2.7 (23–34)	28.2 ± 3.3 (24–34)
Mean follow-up (months)	36.5 ± 8.4 (24–58)	38.8 ± 11.3 (24–58)	41.2 ± 14.2 (24–59)

**Fig. 1:**
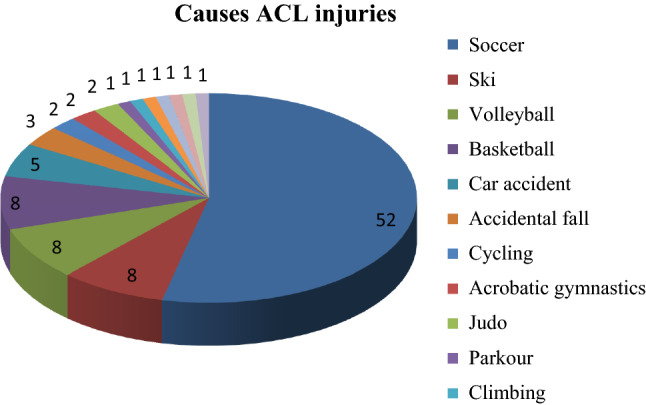
Causes ACL injuries

The cause of ACL disruption was evaluated for each patient (Fig. 1): 91.7% were due to sport-trauma, mainly soccer (53.6%), followed by skiing, volleyball and basketball (8.2% for each sport). Injuries not related to sports were 8.3% (5.2% road accidents and 3.1% accidental falls).

**Fig. 2:**
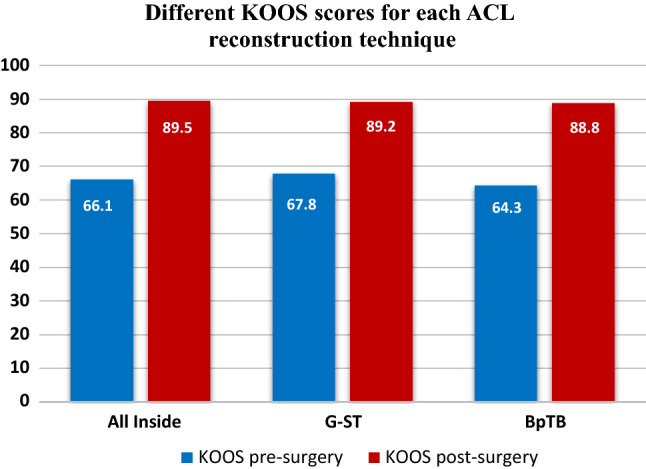
Different KOOS scores for each ACL reconstruction technique

**Fig. 3:**
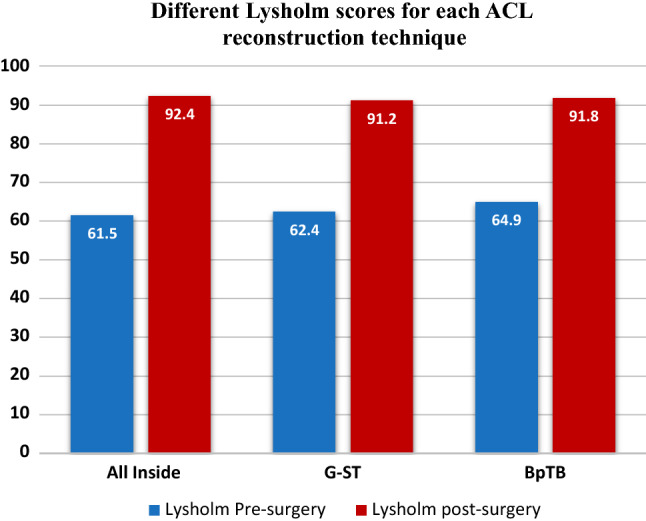
Different Lysholm scores for each ACL reconstruction technique

**Fig. 4:**
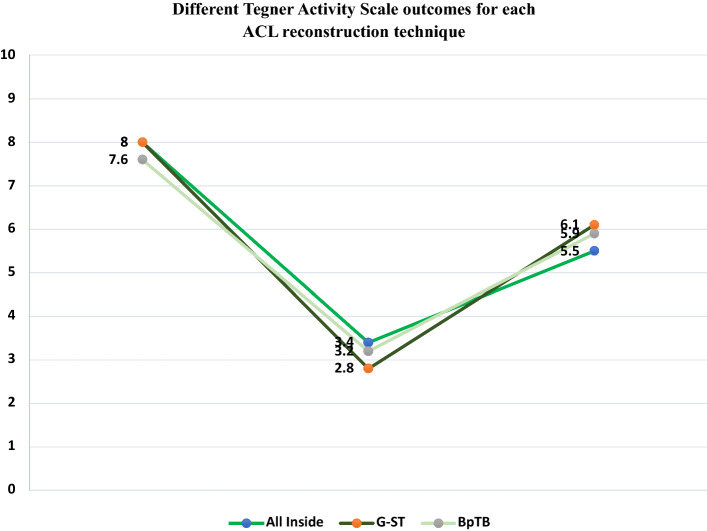
Different Tegner activity scale outcomes for each ACL reconstruction technique

All the descriptive statistics are reported in Tables 1 and 2 and represented in Figs. 2, 3 and 4.

**Table 2:** Clinical outcomes for each surgical technique (AM-HS: antero-medial technique with hamstrings graft; AM-BpTB: antero-medial technique with bone-patellar tendon-bone graft; Pre-op: preoperative value; Post-op: postoperative values)

	All-inside			AM-HS			AM-BpTB		
	Pre-injury	Pre-op	Post-Op	Pre-injury	Pre-op	Post-op	Pre-injury	Pre-op	Post-op
KOOS (Min–Max)		66.1 ± 9.0 (38–73)	89.5 ± 9.6 (63–100)		67.8 ± 8.8 (36–79)	89.2 ± 9.0 (72–100)		64.3 ± 8.5 (34–70)	88.8 ± 7.7 (71–100)
Lysholm (Min–Max)		61.5 ± 9.7 (25–70)	92.4 ± 12.9 (45–100)		62.4 ± 9.2 (27–83)	91.2 ± 9.0 (61–100)		64.9 ± 9.2 (35–80)	91.8 ± 8.4 (68–100)
Tegner (Min–Max)	8.0 ± 1.3 (5–9)	3.4 ± 0.5 (3–4)	5.5 ± 1.8 (3–9)	8.0 ± 1.4 (2–9)	2.8 ± 0.4 (2–4)	6.1 ± 2.2 (2–9)	7.6 ± 1.6 (3–10)	3.2 ± 0.4 (3–4)	5.9 ± 1.9 (3–9)
KT-1000 (Min–Max)			3.1 ± 1.0 (1.3–5.0)			3.3 ± 1.4 (1.3–6.0)			2.5 ± 0.4 (2.0–3.0)

For each group, the results of preoperative score of each questionnaire submitted to the patients were compared with the postoperative ones through Wilcoxon Signed-Rank test (Tegner pre-op vs Tegner post-op; KOOS pre-op vs KOOS post-op and Lysholm pre-op vs Lysholm post-op). A statistical significant improvement was detected (*p* < 0.01) in each score for every surgical technique. Moreover, Tegner scores before injury were studied and compared with Tegner values after surgery: a statistically significant worsening was highlighted (*p* < 0.01) in each surgical group.

The KT-1000 results obtained in both knees in each patient were compared by Wilcoxon Signed-Rank test. The average KT-1000 value for the all-inside group was 3.1 ± 1.0 (1.3–5.0) mm for the operated side and 2.1 ± 0.8 (1.0–4.0) mm for the contralateral one. No statistically significant difference was detected (*p* = 0.16) between those values. In the AM-HS group, the mean KT-1000 value were 3.3 ± 1.4 (1.3–6.0) mm for the operated knee and 2.1 ± 0.8 (0.9–3.8) mm for uninjured one. No statistical significant difference was detected too (*p* = 0.66). Finally, the mean KT-1000 measurement for the AM-BpTB group was 2.5 ± 0.4 (2.0–3.0) mm, 2.2 ± 1.1 (1.0–4.3) mm in the contralateral knee. No statistical difference was found (*p* = 0.15).

Data obtained were compared in order to verify any difference between the three techniques through Kruskal–Wallis test for independent measures. Specifically, the postoperative results of the Tegner scale, KOOS and Lysholm score were compared and no statistical significant difference emerged (*p* = 0.56, *p* = 0.73 and *p* = 0.24, respectively). The results obtained with the KT-1000 were also matched, but no statistical significant difference was detected (*p* = 0.27) between the three surgical techniques.

At the end, the duration of the surgical intervention was recorded (Table 1) and compared between the three surgical methods: a statistically significant difference was highlighted (one-way ANOVA Test for independent measures: *p* < 0.01). All the post hoc correction tests (Tukey’s HSD, Scheffé, Bonferroni and Holm) underlined a statistically significant difference between all-inside durations and AM-BpTB or AM-HS ones (*p* < 0.01); while the difference of the duration between AM-BpTB method and AM-HS technique was no statistically significant (*p* = 0.06).

Although the longer surgical duration of all-inside ACL reconstruction technique than the antero-medial ones, we had no complications in terms of stability, reconstruction failures, deep vein thrombosis (DVT) and infections for all the 157 ACL reconstructions performed. However, only 82 patients (52.2% out of all) restored the pre-injury Tegner level after surgery, regardless of the surgical technique used. In addition, we reported 4 ACL reconstruction failures (2 in the AM-HS group and 2 in the all-inside group; none in AM-BpTB group) due to high energy trauma occurred during sport activity (soccer) at a mean time of 1.6 ± 0.2 (1.3 –1.8) years from surgery in the follow-up period. Those patients had no clinical or radiological signs of instability prior to the second injury. All 4 patients underwent ACL revision surgery.

## Discussion

Anterior cruciate ligament reconstruction is an established and widespread surgical technique. Despite so, uncertainties remain: appropriate timing of surgery, graft selection, fixation methods of the graft, operative techniques and rehabilitation after surgery [23].

The aim of this study was to evaluate the clinical and functional outcomes of patients who underwent all-inside ACL reconstruction at medium-term follow-up and then, to compare this technique with two different ACL reconstruction methods (antero-medial technique using hamstrings or bone-patellar tendon-bone grafts).

Different questionnaires were used to evaluate the patient reported functional results of the ACL reconstruction before and after surgery. The Knee Injury and Osteoarthritis Outcome Score (KOOS) was used: it takes into account symptoms, pain, daily life activities, sports activities and quality of life. Statistically significant improvement in KOOS scores comparing preoperative (average 66.1 ± 9.0) and postoperative (mean 89.5 ± 9.6) periods was found. These results are consistent with Kouloumentas et al. [24] (95.3 ± 3.8) and in Sarraj et al. [25] (89.5 ± 9.6) studies. A further subjective rating scale, Lysholm Knee Questionnaire, was also used. This scale focuses on the residual subjective instability, the pain experienced and in which situation it occurs. The data obtained (61.5 ± 9.7 preoperatively and 92.4 ± 12.9 postoperatively) were similar to those reported in the literature by Volpi et al. [26] (94.9 ± 5.1), Schurz et al. [27] (mean 91.1) and Sarraj et al. [25] (89.9 ± 5.7). The results we obtained reinforce the excellent outcomes shown in the literature and prove the effectiveness of this technique.

The ability to perform physical activities and sports (professional or not) was assessed through Tegner activity scale. Data evaluation showed a significant improvement between pre- and postoperative period (from 3.4 ± 0.5 to 5.5 ± 1.8). However, comparing pre-injury data (mean 8.0 ± 1.3) to the postoperative ones, there was a statistically significant worsening. In fact, the 52.2% of patients returned to the pre-injury sport level. This percentage is similar compared to Ardern et al.’s studies [28, 29]. As the authors described in their systematic review, from 44 to 88% of the patients returned to their preinjury level, and only 55% returned to competitive level sport. Many factors seem to influence the return to sport, but in the literature these are not strongly evident: younger age seems to favor the return to sport as men have greater odds than women, or elite-athletes than non-elite athletes [28].

The KT-1000 allows objective evaluation of the anterior cruciate ligament laxity. Measurements we obtained after surgical reconstruction of the ACL (mean 3.1 ± 1.0 mm) were consistent with major studies in the literature [27, 30, 31]. However, it is difficult to compare our data with the ones reported in the literature: very few works describe the procedure and N force applied during the test.

Our opinion is important to highlight that we did not find difference between the operated knee and the uninjured one (2.1 ± 0.8 mm). Same results were obtained in other techniques we used (AM-BpTB and AM-HS).

Secondary endpoint of the study was designed to evaluate the results of the all-inside method compared to the two antero-medial surgical techniques that used either hamstrings or bone-patellar tendon-bone autologous graft.

Both in subjective and objective evaluation scales, no statistically significant difference emerged between those techniques. Based on the recent Connaughton et al. [6] and Fu et al. reviews [32], our results are consistent with the literature.

All-inside technique allows a precise graft positioning on the femoral and tibial side which brings physiological advantages, crucial to promote revascularization and ligamentization [33]. Further advantages are the bone sparing sockets that result in less postoperative pain, faster postoperative recovery and an important convenience in case of possible revision [7–9].

However, it is important to emphasize the surgical complexity of this technique. Different pitfalls must be known to avoid complications (e.g., a suture passing wires management in a little space under arthroscopy view). That means longer surgical time than other “traditional” procedures [5] especially when those pitfalls are ignored and adverse events occur. Although it is widely demonstrated that complications rate increase with the surgical duration, in our experience, we had no complications as previously described.

### Limitations

This study has some limitations. First, the study setting presents the intrinsic limitations of retrospective design. Secondly, patient selection was based on age, diagnosis and the performed technique. Moreover, we reported a medium-term follow-up and a small sample, especially after stratification. Furthermore, measurements with KT-1000 were not taken in the preoperative period and the actual economic analysis was not considered. Finally, despite clinical analysis was performed by the same surgeon, surgical procedures were executed by two different surgeons leading to a less standardized technique.

### Future directions

ACL reconstruction, according to the results of the present study and recent literature, seems to bring similar clinical result and failure rate regardless of technique used and graft selection. Future direction in the field of ACL reconstruction, consequently, may led towards a more tailored approach that combine reconstruction of ACL to associated procedure (e.g., ALL reconstruction [34], RAMP lesion identification and repair [35] that may strengthen the construct reducing residual laxity, pivot shift and, as a result, reduce failure rate). Further study along this path is needed.

## Conclusions

The present study found good postoperative results in patients treated with all-inside and AM technique at medium-term follow-up. A significant (*p* < 0.05) postoperative improvement of each score (KOOS, Lysholm, Tegner -scores and KT-1000) in all groups was detected. Comparing the results obtained, no statistically significant difference was found between the three techniques (*p* = 0.27). Statistically significant differences were highlighted in surgical duration: all-inside method was the longest (117′), followed by AM-BpTB surgery (101′) and AM-HS technique (87′). According to our data, despite slightly longer operative time required when compared to antero-medial (AM) there was no difference in major complications and graft failure rate.
